# The impact of the rise in atmospheric nitrous oxide on stratospheric ozone: *This article belongs to Ambio’s 50th Anniversary Collection. Theme: Ozone Layer*

**DOI:** 10.1007/s13280-020-01428-3

**Published:** 2020-11-21

**Authors:** Rolf Müller

**Affiliations:** Forschungszentrum Jülich GmbH, Institut für Energie und Klimaforschung (IEK-7), 52425 Jülich, Germany

More than forty years ago *Ambio* published a paper that raised concerns about the increased use of fixed nitrogen as fertiliser in that the resulting increase in nitrous oxide (N_2_O) emissions to the atmosphere could result in a significant reduction of the Earth’s ozone shield (Crutzen and Ehhalt 1977). It presented another case of human activities at the Earth’s surface impacting on the stratospheric ozone layer. Today, it is known that atmospheric N_2_O, which is present in the atmosphere in 2020 at a mixing ratio of 332.8 ppb^1^, is not only important for stratospheric ozone but also constitutes the third most important long-lived greenhouse gas (after CO_2_ and CH_4_).

N_2_O is essentially inert in the troposphere and has no significant sinks at the surface of the Earth. However, when transported to the stratosphere it will be broken down mainly via photolysis at short wavelengths (below 200 nm)

$${\text{N}}_{ 2} {\text{O }} + {\text{ h}}\nu \, \to {\text{ N}}_{ 2} + {\text{ O}}\left( {^{ 1} {\text{D}}} \right) . \left( {\text{R1}} \right)$$

To a lesser extent, N_2_O is also broken down by reaction with O(^1^D)

$${\text{N}}_{ 2} {\text{O }} + {\text{ O}}\left( {^{ 1} {\text{D}}} \right) \, \to {\text{ N}}_{ 2} + {\text{ O}}_{ 2} \left( {\text{R2}} \right)$$

and

$${\text{N}}_{ 2} {\text{O }} + {\text{ O}}\left( {^{ 1} {\text{D}}} \right) \, \to {\text{ 2 NO}} \left( {\text{R3}} \right)$$

where the primary source of O(^1^D) in the atmosphere is the photolysis of ozone (at wavelengths below 320 nm). Reaction R3 constitutes the major source of nitrogen oxides in the stratosphere, but less than 10% of the atmospheric N_2_O is converted to nitrogen oxides (e.g. Ravishankara et al. 2009; Portmann et al. 2012; Fleming et al. 2015).

The breakdown in the stratosphere determines the atmospheric lifetime of N_2_O; the SPARC (Stratosphere-troposphere Processes And their Role in Climate) lifetime assessment estimated the N_2_O lifetime to be 123 years (with a 2-σ range of 104–152 years; SPARC 2013), while more recently Prather et al. (2015) recommend a slightly shorter lifetime of 116 ± 9 years, which is however consistent with the SPARC value within uncertainties.

In the stratosphere, nitrogen oxides (NO and NO_2_) destroy ozone through the following catalytic cycle (Crutzen 1970; Johnston 1971)

$$\begin{aligned} {\text{NO }} + {\text{ O}}_{ 3} \to {\text{ NO}}_{ 2} + {\text{ O}}_{ 2} \left( {\text{R4}} \right) \hfill \\ {\text{NO}}_{ 2} + {\text{ O }} \to {\text{ NO }} + {\text{ O}}_{ 2} \left( {\text{R5}} \right) \hfill \\ \ldots \hfill \\ {\text{net }} {\text{O}}_{ 3} + {\text{ O}} \to {\text{ 2 O}}_{ 2} \left( {\text{C1}} \right) \hfill \\ \end{aligned}$$

Cycle C1 constitutes the dominant catalytic ozone loss cycle in the extrapolar regions (Fig. 1). This cycle is essential for quantifying the stratospheric ozone budget and, consequently, the total column of ozone (e.g. Crutzen et al. 1995; Grooß et al. 1999). At altitudes below 20 km and above 45 km, ozone loss driven by the HO_x_ catalytic cycle dominates ozone loss rates (Fig. 1; Crutzen et al. 1995; Grooß et al. 1999; Müller 2009; Portmann et al. 2012).

**Fig. 1:**
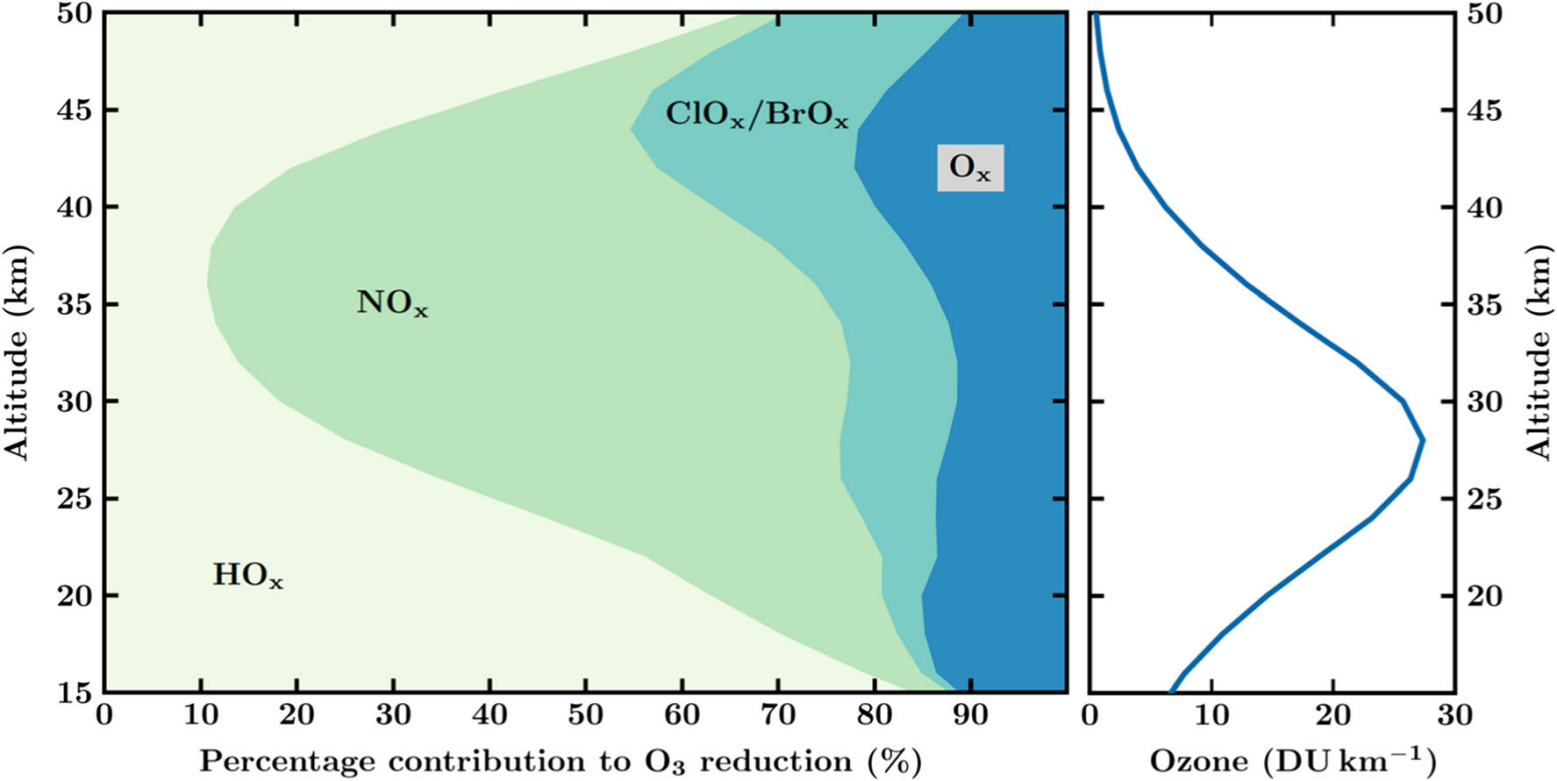
Relative chemical efficiency of catalytic cycles destroying ozone in the stratosphere and the vertical distribution of the ozone concentration (similar to Fig. 2 in Crutzen and Ehhalt, 1977). Data are from a model simulation of the NOCAR model (Portmann et al., 2012) for near global conditions (60°S-60°N) and for levels of source gases for the year 2000. Left hand panel shows the ozone loss rates by chemical family; NO_x_ denotes the catalytic cycle driven by nitrogen oxides (C1); HO_x_ and ClO_x/_BrO_x_ the similar catalytic cycles driven by HO_x_ and ClO_x/_BrO_x_ cycles (see Portmann et al., 2012; WMO 2018), while O_x_ denotes the Chapman (1930) cycle. Right hand panel shows the mean (60°S-60°N) profile of ozone concentrations in units of Dobson units (DU) per kilometre. The Dobson unit is a measure of total column ozone (1 DU = 2.69 × 10^16^ molecules cm^−2^) and DU km^−1^ is a measure of ozone concentration (1 DU km^−1^ = 2.69 × 10^11^ molecules cm^−3^) (Data shown here are courtesy of R. Portmann.)

The paper by Crutzen and Ehhalt (1977) was based on the discussions in the Nobel symposium No. 38, “Nitrogen - An essential life factor and a growing environmental hazard” in 1976, the proceedings of which were published as well in *Ambio* (Bolin and Arrhenius 1977). At that time, many aspects of atmospheric N_2_O were not known and the knowledge of the budget and of sources and sinks of atmospheric N_2_O was very incomplete (e.g. Bolin and Arrhenius 1977; Crutzen and Ehhalt 1977). Some of the N_2_O formation rates proposed in the literature required sources of fixed nitrogen far larger than known. Crutzen and Ehhalt (1977) stated that some “observations showing large spatial and temporal variations of nitrous oxide concentrations in the atmosphere favour the view that there are large sources of nitrous oxide, possibly exceeding 100 Tg (N)/year”; the current estimate for global annual emissions is between 16.1 and 18.7 TgN/year or about 17 TgN/year over the last decade (Thompson et al. 2014, 2019; WMO 2018).

Further, the role of the ocean was unclear, it was stated that “a smaller source or even a sink of N_2_O [at the ocean surface] during larger concentrations of N_2_O should be considered” (Crutzen and Ehhalt 1977). Today, it is established that the oceans are an important natural source of atmospheric N_2_O, with global emissions of about 3.5 TgN/year. In addition to natural emissions, there are currently also anthropogenic emissions of N_2_O from the ocean; the anthropogenic source of N_2_O from the ocean has increased from zero in 1950 to about 1 TgN/year in 2000 (Syakila and Kroeze 2011).

In 1977, there were very few measurements of atmospheric N_2_O with rather large uncertainty ranges; therefore, the global distribution of atmospheric N_2_O and its variability were highly uncertain. In particular, there was only very limited information on the temporal development of N_2_O. A first step forward were measurements of N_2_O between 1976 and 1980 at monitoring stations and aboard research vessels; these measurements showed an annual increase of N_2_O of ~ 0.2% per year and a mean global abundance of N_2_O (in 1978) of ~299.8 ppb (Weiss 1981). The temporal development and the global average mixing ratio of N_2_O were confirmed and extended later by further measurements (e.g. Prinn et al. 1983, 1990).

Determining the absolute calibration for atmospheric measurements of N_2_O accurately is demanding; however consistent atmospheric measurements of the temporal development of N_2_O from independent monitoring networks are available today (Dlugokencky et al. 1994; Saikawa et al. 2014; Prinn et al. 2018; WMO 2018; Dlugokencky et al. 2020; Lan et al. 2020) (Fig. 2). In recent years, N_2_O has been growing relatively steadily, at a rate of about 0.8–1.0 ppb/year (WMO 2018; Lan et al. 2020; see also light blue line in Fig. 2). The historical development of concentrations of N_2_O in the atmosphere is known from measurements in firn and ice cores (Fig. 2, blue line). Measurements in firn provide information on the more recent history (the past ~65 years) and measurements in ice cores are going back 2000 years with a reasonable time resolution (Fig. 2), but can provide information up to 800 000 years back in history (MacFarling Meure et al. 2006; Rubino et al. 2019).

**Fig. 2:**
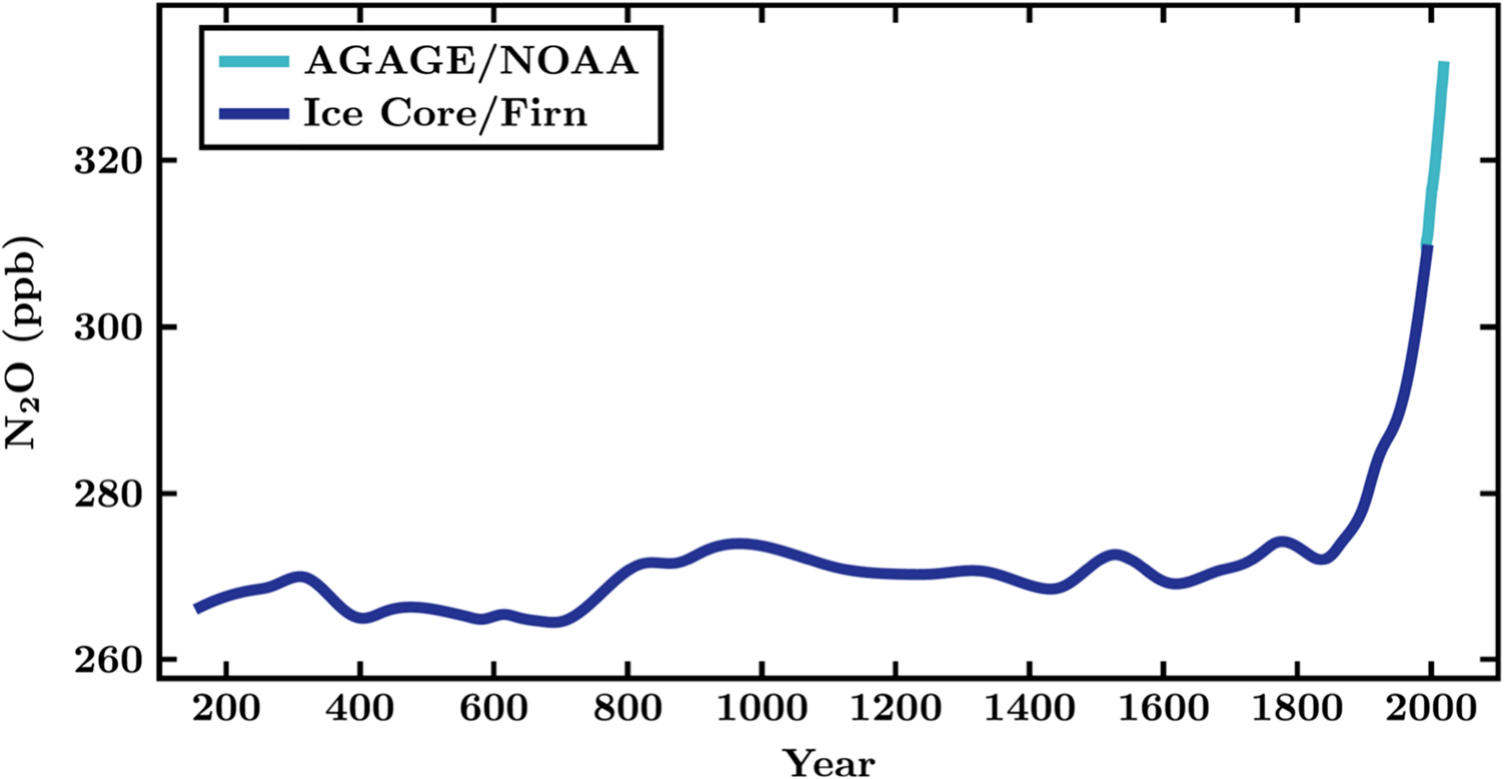
Temporal development of atmospheric concentrations of N_2_O over the past ~2000 years. Historical data are deduced from the Law Dome ice core, more recent data (for the past ~65 years) are deduced from firn air (blue line, MacFarling Meure, et al. 2006; Rubino et al., 2019). The most recent data (after 1995, light blue line) are global mean data from the atmospheric monitoring networks AGAGE (Prinn et al., 2018) and NOAA (Dlugokencky et al., 2020). The recent AGAGE and NOAA records agree so well that they are not distinguished in this figure. Both the AGAGE and the NOAA record continues to today. (The spline fit applied to the ice core and firn data attenuates variations with periods of less than 100 years (MacFarling Meure, et al. 2006; Rubino et al., 2019).) Note that the industrial production of fixed nitrogen (Haber-Bosch process) started in 1913. Data sources: Law Dome Ice Core data for N_2_O (https://data.csiro.au/collections/collection/CIcsiro:37077v1) and data on the recent N_2_O atmospheric mixing ratios from station networks (NOAA/GML: www.esrl.noaa.gov/gmd/ccgg/trends_n2o/ and AGAGE : https://agage.mit.edu/data/agage-data)

In view of the many uncertainties present in 1977, the statements of Crutzen and Ehhalt (1977) were remarkably far sighted. Their paper made a strong point that “increased use of fixed nitrogen as fertilizer might result in a reduction of the Earth’s ozone shield”. A quantification of the fraction of ozone loss caused by anthropogenic N_2_O emissions was however prevented by these uncertainties in 1977. In fact, this environmental problem has been addressed by numerous authors since and is still an area of active research (e.g. Ravishankara et al. 2009; Portmann et al. 2012; Fleming et al. 2015; Butler et al. 2016).

Further, Crutzen and Ehhalt (1977) stated that it “seems possible that present agricultural and waste treatment practices will lead gradually to more N_2_O in the atmosphere by denitrification of the increasing amounts of fixed nitrogen being put in circulation by intensive agricultural methods”. Today it is established that agricultural practices and the use of nitrogen fertilisers have greatly enhanced emissions of N_2_O to the atmosphere (with agricultural emissions amounting to 3.9–5.3 TgN/year in 2010; Syakila and Kroeze 2011; Thompson et al. 2019).

Finally, an important statement by Crutzen and Ehhalt (1977) was that “agricultural use of industrially (or maybe biologically) fixed nitrogen is by no means the only way in which man is interfering with the Earth’s nitrogen cycle. It is for example likely that global N-fixation rates were substantially smaller prior to man’s agricultural transition”. This statement in 1977 was made without the knowledge of the temporal development of N_2_O in the atmosphere (Fig. 2). Today it is known that N_2_O emissions have increased substantially from 10 to 12 TgN/year before the industrial era to ~17 TgN/year in the last decade. Agricultural emissions caused the majority of this increase (emissions of 0.3–1.0 TgN/year in 1850 and 3.9–5.3 TgN/year in 2010), but there is also formation of N_2_O during combustion and industrial processes (Syakila and Kroeze 2011; Thompson et al. 2019).

In the future, because of the success of the Montreal Protocol and its amendments and adjustments in reducing ozone depleting halogen compounds in the atmosphere (WMO 2018), stratospheric ozone in the latter half of the 21^st^ century will be controlled by the temporal development of CO_2_, CH_4_, and N_2_O (Portmann et al. 2012; Butler et al. 2016). Because of the expected increase in atmospheric N_2_O, the NO_x_ driven ozone loss cycle (C1) is expected to be the dominant, anthropogenically driven ozone loss cycle in the foreseeable future (Ravishankara et al. 2009; Portmann et al. 2012). However, because of the considerable uncertainties in emission estimates for N_2_O, an accurate quantification of the anthropogenically caused ozone loss through the NO_x_ cycle (C1) is still challenging (Ravishankara et al. 2009).

Most plants cannot use molecular nitrogen (N_2_). Thus, although N_2_ is by far the most abundant form of nitrogen in the Earth’s atmosphere, the conversion or “fixation” of N_2_ into biologically available compounds is a process fundamental to the maintenance of life on Earth (e.g. Bolin and Arrhenius 1977). Emissions of N_2_O depend on the specifics of the nitrogen fixation processes. An accurate quantification of sectoral emissions of N_2_O (e.g. from agricultural practices, waste management and industrial sources) remains a challenge in the future. The best-known example of industrial nitrogen fixation, the Haber-Bosch process (a method of directly synthesising ammonia from molecular hydrogen and molecular nitrogen) was implemented on industrial scale in 1913. Only the establishment of the Haber-Bosch process made the large-scale, industrial production of synthetic fertiliser possible and in this way likely allowed the rise in the world’s population (e.g. Bodirski et al. 2014). The development of abatement strategies for a reduction of anthropogenic emissions of N_2_O remains therefore a necessary, but not a simple task (Bodirski et al. 2014).

The paper by Crutzen and Ehhalt (1977) ends expressing the hope that it will be possible in the future to “lessen the stress exerted on the Earth’s nitrogen cycle by man’s enormous industrial and agricultural expansion”. Earlier in their paper, the authors also stated that the application of large quantities of fixed nitrogen to agricultural soils will be necessary “to feed adequately the expected world population by the year 2010, at which time we may hope for a halt in further population growth”. Unfortunately, the latter hope has not yet materialised and the anthropogenic stress on the Earth’s nitrogen cycle continues to be a serious issue.

## Footnotes

1 In May 2020, based on the NOAA data web page, see caption of Fig. 2.
